# Cocoa Phenolic Extract Protects Pancreatic Beta Cells against Oxidative Stress

**DOI:** 10.3390/nu5082955

**Published:** 2013-07-31

**Authors:** María Ángeles Martín, Sonia Ramos, Isabel Cordero-Herrero, Laura Bravo, Luis Goya

**Affiliations:** 1 Department of Metabolism and Nutrition, Instituto de Ciencia y Tecnología de Alimentos y Nutrición (ICTAN–CSIC), Madrid 28040, Spain; E-Mails: amartina@ictan.csic.es (M.A.M.); s.ramos@ictan.csic.es (S.R.); isa.cordero@ictan.csic.es (I.C.-H.); lbravo@ictan.csic.es (L.B.); 2 Centro de Investigación Biomédica en red de Diabetes y Enfermedades Metabólicas Asociadas (ISCIII), Madrid 28039, Spain

**Keywords:** antioxidant defences, cocoa flavanols, dietary polyphenols, Ins-1E cells, oxidative biomarkers, type 2 diabetes mellitus

## Abstract

Diabetes mellitus is associated with reductions in glutathione, supporting the critical role of oxidative stress in its pathogenesis. Antioxidant food components such as flavonoids have a protective role against oxidative stress-induced degenerative and age-related diseases. Flavonoids constitute an important part of the human diet; they can be found in most plant foods, including green tea, grapes or cocoa and possess multiple biological activities. This study investigates the chemo-protective effect of a cocoa phenolic extract (CPE) containing mainly flavonoids against oxidative stress induced by *tert*-butylhydroperoxide (*t*-BOOH) on Ins-1E pancreatic beta cells. Cell viability and oxidative status were evaluated. Ins-1E cells treatment with 5–20 μg/mL CPE for 20 h evoked no cell damage and did not alter ROS production. Addition of 50 μM *t*-BOOH for 2 h increased ROS and carbonyl groups content and decreased reduced glutathione level. Pre-treatment of cells with CPE significantly prevented the *t*-BOOH-induced ROS and carbonyl groups and returned antioxidant defences to adequate levels. Thus, Ins-1E cells treated with CPE showed a remarkable recovery of cell viability damaged by *t-*BOOH, indicating that integrity of surviving machineries in the CPE-treated cells was notably protected against the oxidative insult.

## 1. Introduction

Pancreatic β cell failure is a critical metabolic disorder in the development of type 2 diabetes. Decreased viability and dysfunction of β cells would accelerate the diabetic pathogenesis associated with higher mortality. Chronic high glucose exposure would directly increase intracellular ROS generation and deteriorate mitochondrial function to uncouple ATP generation, impairing the glucose-stimulated insulin secretion [1]. Diabetes mellitus in experimental animals and humans is associated with reductions in antioxidants such as ascorbic acid and glutathione, suggesting the critical role of oxidative stress in its pathogenesis [1]. Antioxidant food components have a protective role against oxidative stress-induced degenerative and age-related diseases, cancer and aging [2,3]. Important candidates are plant polyphenols, especially flavonoids, which are naturally occurring compounds widely distributed in vegetables, fruits and beverages such as tea and wine, and possess different biological activities such as antioxidant, anti-inflammatory, antiviral and anti-carcinogenic [2,3].

Another first-rate source of flavonoids is cocoa; in fact, cocoa-derived products are highly consumed in many countries in the European Union and in United States [4]. Cocoa is a rich source of flavonoids, particularly flavanols such as (−)-epicatechin (EC), (+)-catechin, and procyanidins, which are oligomers derived from EC and catechin [5]. Other minor polyphenols have also been identified in cocoa, such as quercetin, isoquercitrin (quercetin 3-*O-*glucoside), quercetin 3-*O*-arabinose, hyperoside (quercetin 3-*O-*galactoside), naringenin, luteolin and apigenin [5]. Cocoa flavanols are potent antioxidants, and their radical scavenging capacity is much higher in cocoa than in black tea, green tea, or red wine [6]. They can be considered as dietary antioxidants and, therefore, as natural products with therapeutic properties or nutraceuticals. Supporting this, numerous *in vitro* and *in vivo* studies have shown that cocoa and its flavanols play a main role as cardiovascular protectors [7], regulators of immune response [8] and have potential preventive roles against tumour processes [9]. Indeed, a cocoa extract mainly containing flavanols, namely cocoa phenolic extract (CPE), has been shown to protect liver cells against an oxidative insult [10] and to up-regulate antioxidant enzymes activity via ERK1/2 pathway to protect against oxidative stress-induced apoptosis in hepatic cells [11]. Additionally, CPE has been reported to prevent TNF-alpha-induced inflammation in colon cells [12]. All these properties indicate that CPE may have remarkable health protective effects in pancreatic beta cell against oxidative stress, benefits which have already been shown for individual cocoa flavanols [13,14,15]. Despite these relevant facts, research on the anti-diabetic properties of flavanols has mostly been focused so far on the galloyl derivatives present in tea [16,17,18].

The study of the effect of dietary compounds on the regulation of antioxidant defence mechanisms may benefit from the use of an established cell culture line such as human Ins-1E. These cells have important biological features of the pancreatic islet beta-cells and have been widely used as a reliable model of beta-cells [17,19]. The aim of the study was to test the potential chemo-protective effect of CPE against oxidative stress chemically induced by a potent pro-oxidant, *tert*-butylhydroperoxide (*t*-BOOH) in cultures of Ins-1E cells. Cell integrity, antioxidant defences and markers of oxidative damage were evaluated to assess the effect of the flavanol in the cellular response to a chemically-induced oxidative stress.

## 2. Materials and Methods

### 2.1. Reagents

*t*-BOOH, glutathione reductase (GR), reduced (GSH) and oxidized glutathione, NADPH, *O*-phthaldehyde (OPT), dichorofluorescin (DCFH), dinitrophenylhydrazine (DNPH), bovine serum albumin (fraction V), gentamicin, penicillin G and streptomycin were purchased from Sigma Chemical (Madrid, Spain). Bradford reagent was from BioRad Laboratories S.A (Madrid, Spain).

### 2.2. CPE Preparation

Natural Forastero cocoa defatted powder (kindly supplied by Nutrexpa, Barcelona, Spain) was used for this study. Soluble polyphenols were extracted as described elsewhere [10]. CPE so obtained was assayed for its *in vitro* antioxidant capacity by the oxygen radical absorbance capacity (ORAC) method and its specific composition additionally analyzed by LC-MS, these results have been previously published and will be briefly summarized in discussion [10].

### 2.3. Cell Culture

Human Ins-1E cells (a gift from Dr. Mario Vallejo, Instituto de Investigaciones Biomédicas “Alberto Sols”, CSIC, Madrid, Spain) were maintained in a humidified incubator containing 5% CO_2_ and 95% air at 37 °C. They were grown in RPMI-1640 medium from Biowhitaker (Lonza, Madrid, Spain) with 11 mM glucose, supplemented with 10% Biowhitaker foetal bovine serum (FBS), 1% Hepes, 1 mM sodium pyruvate, 50 μM beta-mercaptoethanol and 1% of the following antibiotics: gentamicin, penicillin and streptomycin.

### 2.4. CPE and *t*-BOOH Treatment

The different concentrations of CPE, 5, 10 and 20 μg/mL, were diluted in RPMI-1640 culture medium and added to the cell plates for 20 h to test the direct effect of the extract. To induce a condition of oxidative stress, Ins-1E cells were treated with 50 μM *t*-BOOH for 2 h and then tested for ROS production, cell viability, antioxidant defences and carbonyl groups. To evaluate the protective effect of CPE against *t*-BOOH-induced toxicity, concentrations of CPE were diluted in culture medium and added to the cell plates for 20 h; then, the medium was discarded and fresh medium containing 50 μM *t*-BOOH was added for 2 h. The same parameters stated above were evaluated. The selection of the concentrations of CPE to test is explained below (see Discussion).

### 2.5. Evaluation of Cell Viability and ROS Production

Cell viability was determined by using the crystal violet assay [20]. Cells were seeded at low density (10^4^ cells per well) in 96-well plates, grown for 20 h and incubated with crystal violet (0.2% in ethanol) for 20 min. Plates were rinsed with water and 1% sodium dodecylsulfate (SDS) added. The absorbance of each well was measured using a microplate reader at 570 nm. Intracellular ROS were quantified by the DCFH assay using micro plate reader [21]. After being oxidized by intracellular oxidants, DCFH will become dichorofluorescein (DCF) and emit fluorescence. To test for the direct effect of CPE, the DCFH probe was added for 30 min to cells cultured in 24-wells multiwell plates, then the unabsorbed probe was removed and cells were treated with different concentrations of CPE for 2 h and fluorescence determined. To test for the protective effect, cells were treated with different doses of CPE for 20 h, then the DCFH probe was added for 30 min and the unabsorbed probe removed by washing with PBS before being treated with CPE-free medium containing 50 µM *t*-BOOH. After 2 h fluorescence at 485/530 was determined.

### 2.6. Determination of GSH Concentration and GPx and GR Activity

The concentration of GSH and oxidized glutathione was evaluated by a fluorometric assay previously described [21]. The method takes advantage of the reaction of GSH with OPT at pH 8.0 and fluorescence was measured at an emission wavelength of 460 nm and an excitation wavelength of 340 nm. The determination of GPx activity is based on the oxidation of reduced glutathione by GPx, using *t*-BOOH as a substrate, coupled to the disappearance of NADPH by GR [21]. GR activity was determined by following the decrease in absorbance due to the oxidation of NADPH utilized in the reduction of oxidized glutathione [21].

### 2.7. Determination of Carbonyl Groups

Protein oxidation of cells was measured as carbonyl groups content in supernatants according to a published method [22]. Absorbance was measured at 360 nm and carbonyl content was expressed as nmol/mg protein using an extinction coefficient of 22,000 nmol/L/cm. Protein was measured by the Bradford reagent.

### 2.8. Statistics

Statistical analysis of data was as follows: prior to analysis the data were tested for homogeneity of variances by the test of Levene; for multiple comparisons, one-way ANOVA was followed by a Bonferroni test when variances were homogeneous or by Tamhane test when variances were not homogeneous. The level of significance was *p* < 0.05. A SPSS version 19.0 program has been used.

## 3. Results

### 3.1. Effect of CPE on Redox Status of Cultured Ins-1E Cells

**Figure 1:**
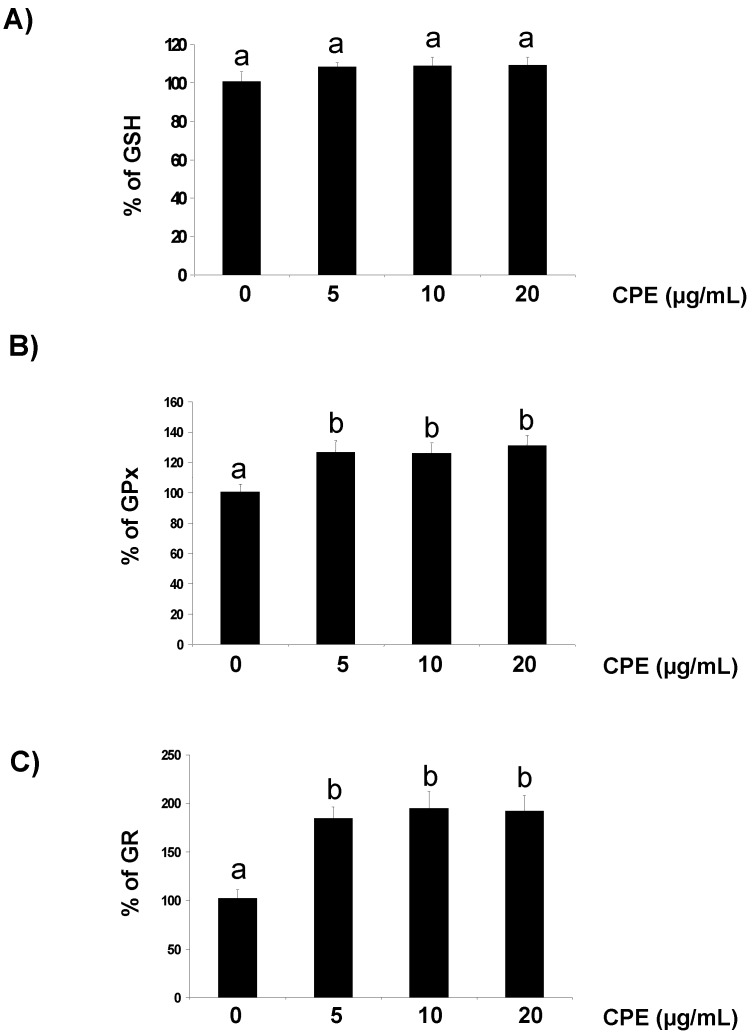
Effect of CPE on GSH concentration and GPx and GR activity. Ins-1E cells were treated with 5–20 μg/mL CPE for 20 h and then washed and collected to test for fluorescent analysis of GSH concentration (**A**) and spectrophotometric assay of GPx (**B**) and GR (**C**) activity. Basal values were 4.1 ± 0.9 nmol/mg protein (GSH), 52 ± 5 mU/mg protein (GPx) and 2.6 ± 0.1 mU/mg protein (GR). Values are means of 5 different samples per condition. Values are expressed as a percent relative to the control condition. Different letters indicate statistically significant differences (*p* < 0.05) among different groups.

In the first part of the present study, Ins-1E cells were treated with 5–20 µg/mL CPE and several parameters related to the cellular redox status and antioxidant response were evaluated. Ins-1E cells treated for 20 h with realistic doses of CPE show no increase in ROS concentration and crystal violet staining after 20 h (Table 1), indicating no cellular stress or damage. Interestingly, treatment with 5–20 µg/mL CPE maintained an unaffected the cellular store of GSH (Figure 1A) and evoked a significant increase in the enzymatic activity of GPx (Figure 1B) and GR (Figure 1C). These results ensure that the Ins-1E cells treated with 5–20 µM CPE are absolutely functional and in favourable conditions to face a stressful challenge.

**Table 1:** Effect of 20 h treatment with noted concentrations of CPE on cell viability and intracellular ROS generation in pancreatic Ins-1E cells.

Condition	Concentration	% Cell Viability	ROS (% of Fluorescence Units)
**C**		100.5 ± 9.6 ^a^	100.6 ± 8.9 ^a^
**CPE**	5 μg/mL	108.3 ± 6.6^ a^	102.3 ± 8.1 ^a^
	10 μg/mL	109.7 ± 9.4 ^a^	110.3 ± 9.2 ^a^
	20 μg/mL	111.4 ± 7.1 ^a^	113.5 ± 9.8 ^a^

^a^ indicates no significant differences among data.

### 3.2. Response of Cultured Ins-1E Cells to a Chemically-Induced Oxidative Stress

**Figure 2:**
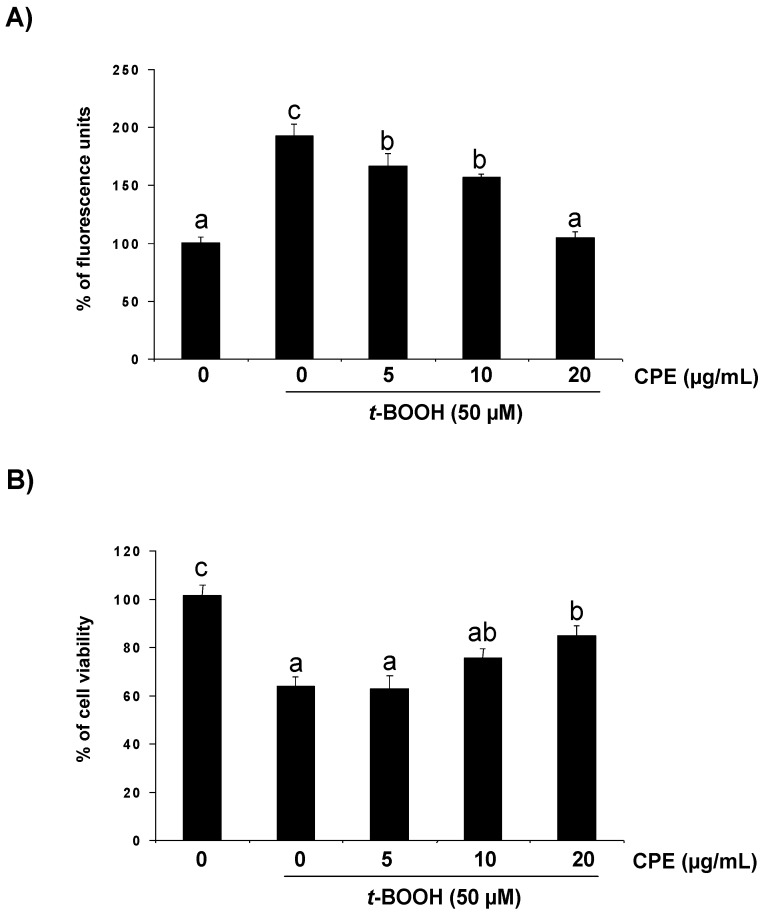
Protective effect of CPE on intracellular ROS generation and cell viability. Ins-1E cells were treated with 5–20 μg/mL CPE for 20 h and then treated with 50 μM *t*-BOOH for 2 h and ROS (**A**) and cell viability (**B**) were determined. Values are means ± SD of 7–8 different samples per condition. Values are expressed as a percent relative to the control condition. Different letters upon symbols indicate statistically different data (*p* < 0.05).

**Figure 3:**
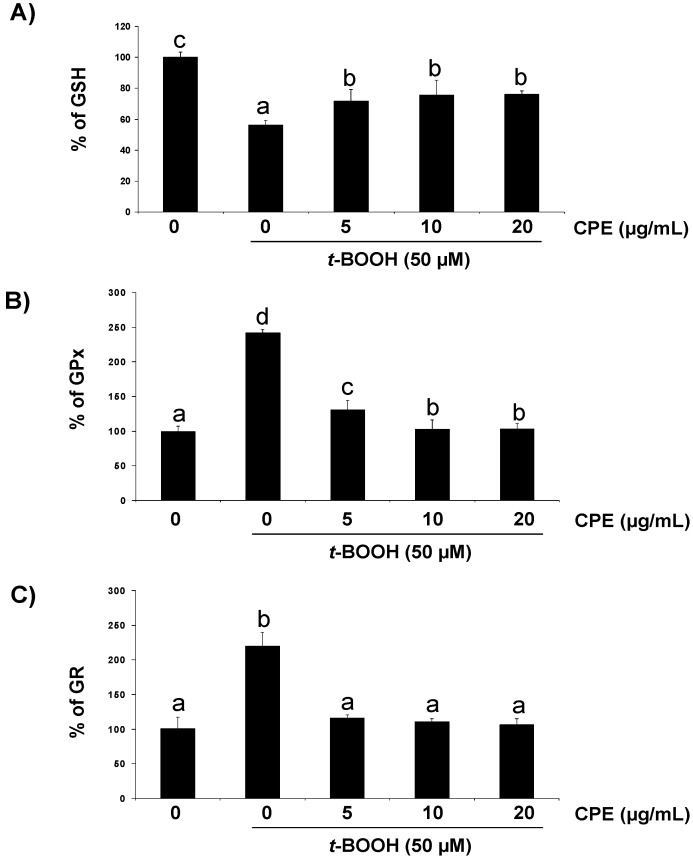
Protective effect of CPE on GSH concentration and GPx and GR activity. Ins-1E cells were treated with 5–20 μg/mL CPE for 20 h and then washed and submitted to 50 μM *t*-BOOH for 2 h prior to assay for GSH (**A**), GPx (**B**) and GR (**C**) to test for the protective effect. Values are means ± SD, *n* = 5. Values are expressed as a percent relative to the control condition. Different letters indicate statistically significant differences (*p* < 0.05) among different groups.

**Figure 4:**
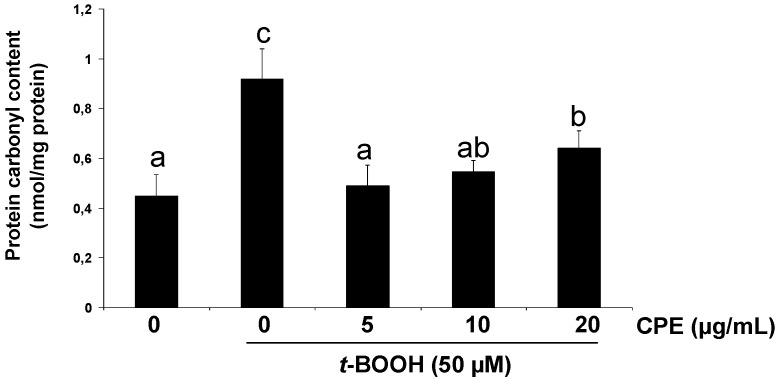
Protective effect of CPE on carbonyl group production. Ins-1E cells were treated with 5–20 μg/mL CPE for 20 h and then treated with 50 μM *t*-BOOH for 2 h and carbonyl groups were determined. Values are means ± SD of 4–5 different samples per condition. Different letters upon symbols indicate statistically different data (*p* < 0.05).

As other organic peroxides, *t*-BOOH can decompose to other alkoxyl and peroxyl radicals in a reaction aided by metal ions that can generate ROS [21]. Thus, 50 µM *t*-BOOH for 2 h enhanced ROS generation (Figure 2A) and cell damage resulting in a remarkably decreased Ins-1E cell viability (Figure 2B). Additionally, important changes in the antioxidant defence system were observed in Ins-1E cells treated with 50 µM *t*-BOOH, *i.e.*, a dramatic decrease of GSH (Figure 3A) and an urgent and acute response of the antioxidant enzymes GPx (Figure 3B) and GR (Figure 3C) to face the oxidative challenge. Consequently, the same stressful treatment induced a significant raise in carbonyl groups proceeding from oxidative damage to proteins (Figure 4). These results confirm a condition of oxidative stress with permanent cell damage in Ins-1E cells treated with 50 µM *t*-BOOH for 2 h.

### 3.3. Protective Effect of CPE on Cultured Ins-1E Cells Submitted to Oxidative Stress

Under these severe oxidative conditions pre-treatment of Ins-1E cell cultures with 5–20 µg/mL CPE significantly reduced the *t-*BOOH-induced ROS production (Figure 2A) as well as partly but significantly prevented cell death (Figure 2B) and GSH depletion (Figure 3A) and completely recovered GPx (Figure 3B) and GR (Figure 3C) activity to pre-stress values. In line with these results, pre-treatment with 5–20 µg/mL CPE returned carbonyl group concentration (Figure 4) to values that were similar to those observed in control unchallenged Ins-1E cells. These results indicate that CPE treatment protects Ins-1E cell integrity and viability.

## 4. Discussion

Diabetes mellitus has been associated with reductions in antioxidants, pointing to a critical role of oxidative stress in its pathogenesis [1]. Cocoa flavonoids are potent antioxidants, and numerous studies have shown that cocoa and its flavonoids play a main role as cardiovascular protectors and have potential preventive roles against oxidative stress-related pathologies [3,7]. In this study cocoa flavanols present in CPE proved to efficiently protect integrity of insulin-secreting Ins-1E cells against a chemically-induced oxidative stress by reducing ROS over-production, recovering altered antioxidant defences and restraining oxidative stress biomarkers.

Characterization of the polyphenolic profile of CPE carried out in a previous study showed that monomeric EC (383 mg/100 g) and catechin (117 mg/100 g) were the major flavanols in the extract, together with appreciable amounts of procyanidins B1 and B2 (133 mg/100 g). Concurrently with flavanols, dimethyl xanthines such as theobromine were present in high amounts and traces of caffeine and theophylline were detected in the extract [10]. The antioxidant capacity of CPE measured by ORAC hydrophilic assay was 620.5 ± 20.3 µmol of Trolox equivalents/g [10]. This value is noticeably higher than those reported for other extracts from fruits and nuts with well-known antioxidant activity such as strawberry (202 µmol TE/g d.m.) and walnuts (154 µmol TE/g d.m.) [23]. Regarding cocoa derivatives, the ORAC value for the CPE was lower than that found in baking chocolate (1040 µmol TE/g) yet much higher than the values reported for milk chocolate candy bars (81.7 µmol TE/g) [24]. This remarkable antioxidant capacity makes the cocoa polyphenolic fraction an interesting candidate for cellular chemoprotection.

Although cocoa flavanols may have potent antioxidant effects *in vitro* and *in vivo*, both in cell culture and live animals, elevated doses of this dietary foodstuff can also act as pro-oxidant in cell culture systems and evoke cellular damage [25,26]. Therefore, it is necessary to ensure that no direct cell damage is caused by reasonable concentrations of the tested antioxidant before aiming for its protective effect [2]. Concentrations of CPE around 5–10 µg/mL were effective in previous experimental conditions [10,11]. Additionally, the concentration range between 5 and 20 µg/mL selected for this study is not far from realistic in order to evaluate the effect at the physiological level; these doses of the cocoa extract contain an equivalent to 0.15 to 0.6 µM flavanols, mainly monomeric catechin and EC and dimeric procyanidins. In line with this, steady-state concentrations of 0.2–0.4 µM EC in humans after ingestion of 80 g of chocolate [27] and 25 g of semisweet chocolate chips [28] have been reported. However, when dealing with a metabolic pathology such as type 2 diabetes it is necessary to point out that beneficial effects of some food componentes may be counteracted by the negative effects of a positive energy balance and, in this line, other sources of flavanols such as tea could be of preference over cocoa/chocolate.

In this study none of the CPE concentrations selected altered ROS production and evoked cell damage in Ins-1E cells after 20 h. Moreover, treatment of Ins-1E cells with physiological concentrations of the extract preserved their GSH store, maintaining the cell in steady conditions to face a potential oxidative challenge.

In addition to their antioxidant capacity by directly scavenging intracellular ROS, flavanols have been recently shown to provide a parallel protection by enhancing the activity of a number of protective enzymes [11,29]. In this line, we have reported that in doses ranging from 1.5 nM to 1.5 µM cocoa flavanols up-regulate antioxidant enzyme activity via extracellular regulated kinases pathway in liver cells [11] and, more recently, cocoa procyanidin B2 induced detoxificant enzyme glutathion-*S*-transferase protein concentration to protect human colonic cells against oxidative stress [30]. GPx catalyzes the reduction of peroxides and is suggested to act as a barrier against hydroperoxide attack, whereas GR is implicated in recycling oxidized glutathione back to reduced glutathione [11]. Therefore, the function of glutathione-dependent enzymes, which participate in the defence against hydrogen peroxides and superoxides, is essential to prevent the cytotoxicity of ROS. In the present study we have found that the same extract evokes a substantial increase in GPx and GR activity in Ins-1E cells. This finding should have a relevant impact on Ins-1E cells due to the low antioxidant enzymes gene expression in pancreatic tissue as compared to other tissues [1]. The results above indicate that CPE-treated Ins-1E cells are in favourable conditions to face the increasing generation of ROS induced by the potent pro-oxidant *t*-BOOH and consequently to maintain cell function and escape death.

Treatment of cells with the strong pro-oxidant *t*-BOOH is an excellent model of oxidative stress in cell culture systems from different origin such as liver (HepG2) [10,21,31,32], colon (Caco-2) [33] and pancreatic beta cell (Ins-1E) [34]. A significant increase in ROS was observed in Ins-1E cells treated for 2 h with 50 µM *t*-BOOH. In parallel, antioxidant defences were also dramatically affected by the oxidative challenge, evoking a significant depletion in GSH and a remarkable increase of GPx and GR activities to cope with the augmenting ROS. As a direct consequence of the altered redox status a two-fold increase in the concentration of the biomarker of oxidative damage to proteins, carbonyl groups, was found. All these changes resulted in a severe decrease in cell viability, indicating that 50 µM *t*-BOOH evoked a condition of oxidative stress and cell damage in cultured Ins-1E. In this model, the *t*-BOOH-induced increase in ROS generation was significantly prevented in cultured cells pre-treated with CPE. These results with the cocoa extract are in line with those reporting a protective effect of a tea flavanol, epigallocatechin gallate [19] and EC [34] in Ins-1E cells against an induced oxidative effect; this suggests that the ROS generated during the period of oxidative stress were more efficiently quenched in cells pre-treated with the flavanol-rich cocoa extract. This should be considered as the first beneficial effect of CPE on stressed Ins-1E cells.

GSH plays an important role in protection against oxidative stress as a substrate in glutathione peroxidase-catalysed detoxification of organic peroxides, by reacting with free radicals and by repairing free-radical-induced damage through electron-transfer reactions [35]. In this study, the remarkable decrease in the concentration of GSH induced by an oxidative condition in Ins-1E cells was partly prevented by pre-treatment with CPE evoking a significant recovery of GSH. Since onset of diabetes mellitus in experimental animals and humans has been associated with reductions in antioxidants such as GSH [1,36], the effect of CPE maintaining GSH concentration above a critical threshold while facing a stressful situation represents a decisive advantage for pancreatic beta cell survival.

Induction of GPx and GR is an essential mechanism of the cell defence against oxidative insults and consequently plays a major role to overcome ROS production in the presence of *t*-BOOH [10,21,30,31,32]. An increase in GPx activity faces ROS overproduction at the expense of GSH, the decrease of which is recovered by an enhanced GR activity. However, a rapid return of antioxidant enzyme activities to basal values once the challenge has been surmounted will place the cell in a favorable condition to deal with a new oxidative insult. In this study, pre-treatment of Ins-1E cells with CPE managed to prevent the long-lasting increase in the activities of GPx and GR induced by oxidative stress. This phenomenon, unreported to date in pancreatic beta cells, is consistent with previous results with individual flavonoids such as quercetin [21], green tea catechins [37] as well as the same CPE [10,11] in hepatic HepG2 cells.

Carbonyl groups are considered as consistent markers of oxidative damage to proteins, a crucial event in the development of cellular toxicity [22,38]. The significant increase in the cellular concentration of carbonyl groups during oxidative stress induced by *t-*BOOH in Ins-1E cells indicated extensive damage to cellular proteins. Pre-treatment of cells with 10–20 µM CPE significantly reduced the level of carbonyl groups demonstrating a smaller degree of protein oxidation in response to the stressful situation. The cyto-protective effect of CPE on a marker of oxidative damage has previously been reported [10] and a comparable protection on oxidative markers has been observed with other dietary compounds including plant polyphenols such as quercetin [21], EC [31], tea catechins [37,39] and olive oil hydroxytyrosol [40] in cultured liver cells. Interestingly, a specific chemo-protective effect of EC on pancreatic beta cells in culture has recently been suggested [34].

Thus, the protective mechanism of CPE on Ins-1E cells submitted to a severe oxidative stress can be illustrated in terms of regulation of the cellular redox status, *i.e.*, CPE decreases ROS production and reduces the necessity of peroxide detoxification through GPx and of GSH repletion from oxidized glutathione through GR. Additionally, decreased ROS level reduces oxidative damage to proteins resulting in mitigated cell death. Interestingly, EC is the major flavanol of cocoa, suggesting that most of the protecting effect of CPE both in liver and pancreatic cells may be due to EC. In agreement with this result, a protective effect of EC on hepatic HepG2 [20,31,41], colonic Caco-2 [33] and pancreatic Ins-1E [34] cell viability submitted to *t*-BOOH has been reported.

In addition to flavanols, CPE contains a comparable quantity of theobromine, a dimethylxanthine widely found in plants, especially in cocoa. Since theobromine has shown a significant inhibitory effect on the inflammatory process in epithelial cells [42], the potential participation of the dimethylxanthine to the protection against oxidative stress was tested in a previous study [10]. The results unequivocally showed that the contribution of theobromine to the protective capacity of CPE may be considered negligible and, therefore, the stress-preventive effect is mostly provided by the flavonoid fraction.

The search for natural compounds, preferably found in a regular diet, has received wide attention as a preventive, rather than curative, approach in order to delay the appearance of diabetic complications. The anti-diabetic effect of EC was first reported by Chackravarthy and colleagues back in 1982 [13], although contradictory results were also rapidly exposed [43]. We have recently demonstrated that EC improves insulin signalling and repress glucose production via AKT and AMPK in hepatic cells [44]. Furthermore, we have just observed for the first time that EC undoubtedly induces glucose-stimulated insulin secretion in cultured Ins-1E cells preventing or delaying a potential beta cell dysfunction [34]. These results agree with recent reports showing that dietary EC promotes a longer lifespan in obese diabetic mice [14] and also agrees with other reports showing the beneficial effects of cocoa and green tea flavanols on blood glucose regulation in obese diabetic adults [15].

## 5. Conclusions

Consequently with the preservation of the antioxidant defence system, Ins-1E cells treated with CPE showed a remarkable attenuation of cell damage after being submitted to stress. These results indicate that integrity of surviving machineries in the CPE-treated Ins-1E cells was notably protected against the oxidative insult. This study demonstrates the chemo-protective effect of a flavanol-containing foodstuff such as cocoa, which likely plays a role in the protection afforded by fruits, vegetables and plant-derived beverages against diseases such as type 2 diabetes, for which excess production of ROS has been implicated as a causal or contributory factor. Since these results obtained in cultured cells cannot be directly extrapolated to an *in vivo* situation, experiments with live animals are currently in process.
